# On Uræmia

**Published:** 1861

**Authors:** 


### ON URÆMIA.

By PROF. JAKSCH.

*Vierteljahrschrift fur die praktische Heilkund.* xvii. Jahrgang., 1860.

The author of this paper holds that there are two varieties of uræmia which should be carefully distinguished; one being caused by the decomposition of urine and absorption of carbonate of ammonia into the blood (ammonæmia), the other being the variety which accompanies Bright's disease of the kidneys. He has seen the former occur under the following circumstancces:

1. In torpor and paralysis of the bladder.
2. In dilatation of the pelvis and calices of the kidney in consequence of ureters being blocked up.
3. In renal abscess, renal tuberculosis and saculated kidneys.

The following are the main differences characterizing the two forms of uræmir; we shall, to save circumlocution, use the word ammonæmia as the name of the one, and Bright's uræmia as the name of the other:

1. In advanced ammonaemia the urine discharged from the bladder manifests a strong ammoniacal odor, which Prof. Jaksch has never noticed in any stage of Bright's uraemia.
2. Dropsical symptoms, either acute and febrile, or chronic and afebrile, have not been observed in ammonaemia.
3. Advanced ammonaemia is characterized by persistent dryness of the mucous membrane covering the mouth and fauces, as if every particle of moisture has been removed by blotting-paper; the membrane looks dry and shining, and the dryness even extends to the mucous membrane of the nose, the conjunctiva, and even to the chordae vocales; these symptoms do not occur in Bright's uraemia.
4. The distinctly ammonaical odor of the air exhaled, and of the cutaneous secretions of patients affected with ammonæmia, does not occur in Bright's disease.
5. Patients suffering from ammonaemia always show a marked dislike to meat, and especially brown meats, even if their affections have not advanced very far; a feature rarely seen in the other variety.

6. Prof. Jaksch has never observed in Bright's disease the violent intermittent rigors, stimulating intermittent fever, which occur in ammonaemia.

7. In none of the cases of ammonaemia were convulsive o epileptiform attacks, nor croupy or diphtheretic exudations noticed.

8. Disturbed vision, as produced in Bright's disease by exudation on the retina, does not appear to take place in ammonaemia.

9. Chronic ammonaemia is characterized by a uniformly pale and sallow complexion, and by gradually increasing emaciation; very acute and advanced ammonaemia is associated with very rapid wasting of features, and muscular debility amounting to paralysis.

10. In all cases of ammonaemia which ran a rapid course there was vomiting, with concurrent or consequent diarrhœa; in chronic ammonaemia both phenomena were often entirely absent, or only occurred temporarily.

11. In ammonaemia, whether acute or chronic, Professor Jaksch has always seen death occur after sopor, varying in duration from several hours to several days.

The author of this valuable and interesting paper gives numerous cases illustrative of his views, and enters very fully into the various questions connected with diagnosis and treatment, for which we are unable to make room.—*South. Med. and Surgical Journal.*

